# Neuroprotective Effect of SCM-198 through Stabilizing Endothelial Cell Function

**DOI:** 10.1155/2019/7850154

**Published:** 2019-11-11

**Authors:** Qiu-Yan Zhang, Zhi-Jun Wang, Lei Miao, Ying Wang, Ling-Ling Chang, Wei Guo, Yi-Zhun Zhu

**Affiliations:** ^1^Yantai Institute of Materia Medica, Yantai Branch, Shanghai Institute of Materia Medica, Chinese Academy of Sciences, China; ^2^Shanghai Key Laboratory of Bioactive Small Molecules, Department of Pharmacology, School of Pharmacy, Fudan University, 826, Zhangheng Road, Pudong New District, Shanghai 201203, China; ^3^State Key Laboratory of Quality Research in Chinese Medicine and School of Pharmacy, Macau University of Science and Technology, Macau, China; ^4^Department of Pediatric Surgery, Guangzhou Institute of Pediatrics, Guangzhou Women and Children's Medical Center, Guangzhou Medical University, Guangzhou, 510623 Guangdong, China; ^5^Department of Pharmacology, University of California, Davis, USA

## Abstract

Leonurine, also named SCM-198, which was extracted from *Herba leonuri*, displayed a protective effect on various cardiovascular and brain diseases, like ischemic stroke. Ischemic stroke which is the leading cause of morbidity and mortality, ultimately caused irreversible neuron damage. This study is aimed at exploring the possible therapeutic potential of SCM-198 in the protection against postischemic neuronal injury and possible underlying mechanisms. A transient middle cerebral artery occlusion (tMCAO) rat model was utilized to measure the protective effect of SCM-198 on neurons. TEM was used to determine neuron ultrastructural changes. The brain slices were stained with Nissl staining solution for Nissl bodies. Fluoro-Jade B (FJB) was used for staining the degenerating neurons. In the oxygen-glucose deprivation and re-oxygenation (OGD/R) model of bEnd.3 cells treated with SCM-198 (0.1, 1, 10 *μ*M). Then, the bEnd.3 cells were cocultured with SH-SY5Y cells. Cell viability, MDA level, CAT activity, and apoptosis were examined to evaluate the cytotoxicity of these treatments. Western blot and immunofluorescent assays were used to examine the expression of protein related to the p-STAT3/NOX4/Bcl-2 signaling pathway. Coimmunoprecipitation was performed to determine the interaction between p-STAT3 and NOX4. In the transient middle cerebral artery occlusion (tMCAO) rat model, we found that treatment with SCM-198 could ameliorate neuron morphology and reduce the degenerating cell and neuron loss. In the *in vitro* model of bEnd.3 cell oxygen-glucose deprivation and reoxygenation (OGD/R), treatment with SCM-198 restored the activity of catalase (CAT), improved the expression of Cu-Zn superoxide dismutase (SOD1), and decreased the malondialdehyde (MDA) production. SCM-198 treatment prevented OGD/R-induced cell apoptosis as indicated by increased cell viability and decreased the number of TUNEL-positive cells, accompanied with upregulation of Bcl-2 and Bcl-xl protein and downregulation Bax protein. The results were consistent with SH-SY5Y cells which coculture with bEnd.3 cells. The forthcoming study revealed that SCM-198 activated the p-STAT3/NOX4/Bcl-2 signaling pathway. All the data indicated that SCM-198 protected against oxidative stress and neuronal damage in *in vivo* and *in vitro* injury models via the p-STAT3/NOX4/Bcl-2 signaling pathway. Our results suggested that SCM-198 could be the potential drug for neuroprotective effect through stabilizing endothelial cell function.

## 1. Introduction

Stroke is one of the leading cause of morbidity and mortality worldwide [1], owing to its incredibly short therapeutic time window and fewer effective emergency medicines, tissue-type plasminogen activator (tPA) serving as priority therapeutic drug in ischemic stroke, with only 10% patients of which applicable to this therapy [2]. Clinically speaking, stroke could be categorized into two types: around 85% of ischemic stroke and hemorrhagic stroke which includes intracerebral bleeding and subarachnoidal bleeding accounting for 10% and 3%, respectively [3]. Meanwhile, in the ischemic stroke, secondary damage led by reperfusion will worsen prognosis including a breakdown of blood-brain barrier (BBB), inflammation, oxidative stress, excitotoxicity, and finally irreversible neuronal damage [4].

NADPH oxidases (NOX) are one kind of the main sources of ROS and the only kind of enzyme known that has ROS formation function solely [5]. In mammals, the NOX family includes seven members: NOX1 to NOX5, dual oxidase- (Duox-) 1, and Duox-2 [6–8]. Among NOX, NOX4 appears mostly as a target for ischemia-reperfusion (IR) therapy [9, 10] because it is induced under hypoxia in various cell and tissues making it seem to be the most possible key point of IR injury [11]. In addition, recent researches demonstrated that NOX4 exerted the protective effect against blood-brain barrier breakdown, oxidative stress, and neuronal apoptosis during ischemic stroke [12, 13].

Research revealed that the activated signal transducers and transcription 3 (STAT3) is involved in the protection against cerebral ischemic reperfusion injury [14–16]. Previous studies investigated that activated STAT3 in stroke model could promote numerous genes which play a protective effect on neural injury and repair [17, 18]. Further experiments revealed that the regulation of the STAT3 signaling pathway could prevent neuroapoptosis [19]. However, the further mechanism of the downstream regulators is unclear. On the contrary, there also some other different results which reveal that blocking the STAT3 pathway could improve cerebral recovery and neurological outcomes [20]. Therefore, the rigid contribution of activated STAT3 after stroke remains incompletely explored.

*Herbaleonuri*, also called Chinese Motherwort or Siberian Motherwort, is found in China, central Europe, Scandinavia, and Russia and has been documented for treatment of vaginal bleeding, dystocia, retained fatal membranes, bruising, metrorrhagia, metrostaxis, hemuresis, and some other diseases. Leonurine (C_14_H_21_N_3_O_5_), extracted from the leaves of *Herbaleonuri*, also named SCM-198, was reported to be protective in cardio cerebral vascular diseases. Our previous results firstly provide the evidence that SCM-198 could prevent cardiac fibrosis and activate cardiac fibroblasts partly through modulation of the NOX4-ROS pathway [21]. And our investigation found that SCM-198 could maintain the BBB integrity by regulating the HDAC4/NOX4/MMP-9 tight junction pathway [22–25]. SCM-198 may directly inhibit the overactivated microglia, maintain their ramified morphology, and decrease proinflammatory cytokines via the NF-*κ*B and JNK pathways in microglia and A*β*1-40-injected SD rats [26]. Therefore, we investigated the protective effect of SCM-198 on neuron and microvascular endothelial cells in both tMCAO rat model and OGD/R *in vitro* model and put forward new mechanisms that contribute to the protective effects of SCM-198 via the STAT3/NOX4/Bcl-2 pathways.

## 2. Materials and Methods

### 2.1. Animal Model and Treatment

All the experimental protocol was approved by the institutional ethical committee with internationally accepted ethical standards. Protocols and animal handling were performed in accordance with the guidelines of the National Institutes of Health *Guide for the Care and Use of Laboratory Animals*. Male Sprague-Dawley (SD) rats were supplied by the laboratory animal center, Fudan University. Rats weighing 180-220 g were housed with enough food and water under diurnal lighting condition.

Briefly, we performed the surgery as described previously [27]. All the animals mentioned above were randomly divided into five groups: control operation group, tMCAO group treated with saline, edaravone- (3 mg/kg/day) treated group, and SCM-198 (15 mg/kg/day in normal saline) treatment groups that were posttreated (0.5 h and 2 h after operation). All the drugs were given through tail vein injection once daily for 3 times.

### 2.2. Transmission Electron Microscopy (TEM)

TEM was used to determine neuron ultrastructural changes. All of the ultrathin sections were examined with a Jeol JEM 1200 EX (Jeol Ltd., Tokyo, Japan) transmission electron microscope. An investigator blinded to the study protocol examined tissues [28].

### 2.3. Tissue Prepared

After three days of treatment, the rats (*n* = 6) were anesthetized with pentobarbital sodium (50 mg/kg), then perfused with 0.9% saline and subsequently with 4% paraformaldehyde in PBS. The brains were removed and postfixed over 12 h in the same aldehyde fixative solution, then immersed in 15% and 30% sucrose solution over 6 days at 4°C. The brains were sectioned at 20 *μ*m which were used for the next experiments [29].

### 2.4. Nissl Staining

Brain slices described above were stained with Nissl staining solution (Beyotime) for 20 min. The slices were dehydrated in 70%, 95%, and 100% ethanol, cleared in xylene, then covered with neutral resin. Five sections were selected from each rat and three images for cortex and striatum, respectively. The images were analyzed by ImageJ.

### 2.5. Fluoro-Jade B (FJB) Staining

FJB was used for staining the degenerating neurons. Brain sections described above were stained according to Liu et al. [30].

### 2.6. Immunofluorescent Staining

Immunofluorescence was assessed as described earlier [31, 32]. Coronal brain slices described above were blocked and incubated in polyclonal rabbit anti-NeuN antibody (Abcam, 1 : 500) overnight in 4°C, followed by Alexa Fluor 488-conjugated goat anti-rabbit IgG (1 : 1000, Life Technologies) and counterstaining with DAPI. Fluorescence staining was viewed with a laser scanning confocal microscope (Zeiss, Oberkochen, Germany).

### 2.7. bEnd.3 Cell Culture and Treatment

Mouse bEnd.3 cells were bought from the American Type Culture Collection (ATCC). Cells were cultured according to our previous method [25].

To mimic ischemic-like conditions *in vitro*, bEnd.3 cells were exposed to OGD and reperfusion as we described previously [33]. In brief, the cells were washed with PBS then replaced with glucose-free medium (Invitrogen). The cells were placed in a BioSpherix incubator chamber (ProOx C21, USA), which was flushed with 95% N_2_ and 5% CO_2_ for 6 h then transferred to 95% air, 5% CO_2_, and continued to be cultured in the glucose-containing medium for 4 h each time. The cells were divided into five groups: control, OGD, and cells treated with SCM-198 (0.1 *μ*M, 1 *μ*M, and 10 *μ*M) 2 h before OGD. The inhibitors were added 1 h before OGD until the end of the experiment.

### 2.8. SH-SY5Y Cell Culture and Coculture with bEnd.3 Cells

SH-SY5Y cell lines were purchased from the American Type Culture Collection. SH-SY5Y cells were cultured with Dulbecco's modified Eagle's medium (DMEM, HyClone, USA) containing 10% fetal bovine serum (FBS, Gibco, USA) and 100 *μ*g/mL penicillin/streptomycin (Gibco) and cultured at 37°C containing 5% CO_2_ and 95% O_2_.

The coculture system was set up according to a previous study with some modifications [34]. After coculture for 24 h, the SH-SY5Y cells were washed with PBS then replaced with glucose-free medium (Invitrogen). The cells were placed in a BioSpherix incubator chamber (ProOx C21, USA), which was flushed with 95% N_2_ and 5% CO_2_ for 9 h then transferred to 95% air, 5% CO_2_, and continued to be cultured in the glucose-containing medium for 2 h each time.

### 2.9. MTT and Lactate Dehydrogenase (LDH) Assay

Cell viability was determined by the mitochondrial-dependent reduction of MTT (3-[4,5-dimethylthiazol-2-yl]-2,5-diphenyl tetrazolium bromide) to formazan by adding 10 *μ*L of the MTT agent (5 mg/mL; Sigma-Aldrich) to cells in the plates [35].

LDH activity was detected using the LDH activity assay kit according to the manufacturer's instructions.

### 2.10. The Measurement of the Level of MDA and the Activity of CAT

Lipid peroxidation is quantified by measuring the level of malondialdehyde (MDA) assay kit (Byotime). The catalase activity (CAT) was determined following the manufacturer's instructions (Beyotime).

### 2.11. TUNEL

To measure the cell apoptosis after OGD/R insult, we counted the TUNEL- (terminal deoxynucleotidyl transferase-mediated dUTP-biotin nick-end labeling-) positive cells which were determined by a cell death detection kit, according to the manufacturer's protocol (Biotool).

### 2.12. Coimmunoprecipitation

Coimmunoprecipitation was carried out as described previously [36]. Briefly, bEnd.3 cells were subjected to OGD treatment and reperfusion, then lysed on ice in RIPA buffer. After preclearing with normal IgG, cell lysates (0.5 mg of protein) were incubated overnight at 4°C with 2 *μ*g of anti-NOX4 (Proteintech, 1 : 100) and anti-p-STAT3 (CST, 1 : 100), followed by precipitation with 20 *μ*L of protein A/G Plus-Agarose (Santa Cruz Biotech.) for 1 h at 4°C. The precipitated complexes were used for western blot analysis, as described below.

### 2.13. Western Blot

Western blot analyses were performed as previously described [36, 37].

Each membrane was incubated with specific antibodies as follows: Bcl-xl (Cell Signaling Technology, 1 : 1000), Bcl-2 (Cell Signaling Technology, 1 : 1000), Bax (Cell Signaling Technology, 1 : 1000), SOD1 (Cell Signaling Technology, 1 : 1000), NOX4 (Proteintech, 1 : 1000), STAT3 (Cell Signaling Technology, 1 : 1000), p-STAT3 (Cell Signaling Technology, 1 : 1000), Akt (Cell Signaling Technology, 1 : 1000), and p-Akt (Cell Signaling Technology, 1 : 1000). To measure the expression of each protein, the relative intensity was calculated by comparing the intensity of GAPDH using densitometry.

## 3. Results

### 3.1. The Protection of SCM-198 on Neuron Morphology after Ischemic Stroke

**Figure 1:**
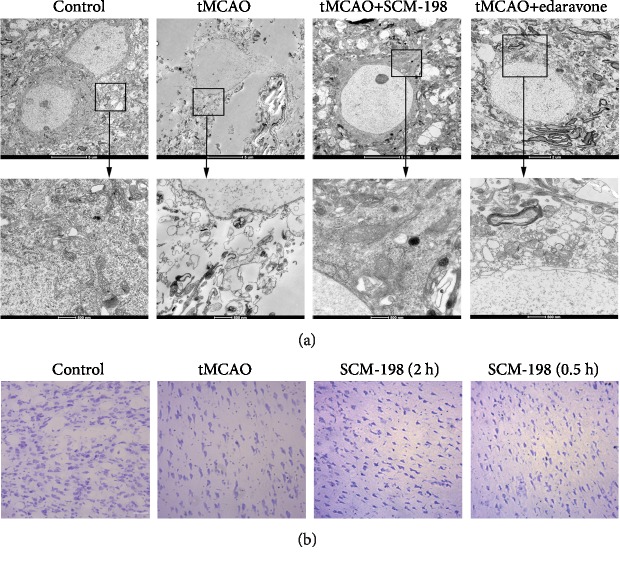
The protection of SCM-198 on neuron morphology after ischemic stroke. (a) The representative TEM of neurons in the peri-ischemic region in the tMCAO model. SCM-198 diminished the changes in neuron morphology after I/R injury. Scale bar = 5 *μ*m and 500 nm, *n* = 3. (b) Representative pictures of coronal sections from the ischemic rat brain stained with Nissl staining. SCM-198 reduced cell shrinkage and empty spaces. Scale bar = 20 *μ*m (*n* = 5).

As we know, reperfusion can cause secondary brain injury, including irreversible neuron losses, injury, and degeneration. According to a previous research, we hypothesized whether SCM-198 exerts the effect on neurons in the tMCAO model. Firstly, we investigated brain tissue ultrastructural conditions. Three days after tMCAO operation, large vacuoles and lysosomes appeared in the cytoplasm. Nearly all of the mitochondria in the model group showed ultrastructural pathological changes and most of them were swollen. We could hardly find normal neurons in this group (Figure 1(a)). SCM-198 treatment groups revealed less intercellular edema, better neuron ultrastructure, and better mitochondrial protection than the tMCAO-insulted group. Well-protected neurons and slight dendritic swelling in 0.5 h post operation treatment groups demonstrated great amelioration after SCM-198 treatment. In the 0.5 h post operation treatment with edaravone group, neurons were swelling and with less dense cytoplasm compared with normal neurons. Mitochondrial accumulation occurred which implicated oxidative stress in the insulted region. We next measured the neural cell loss in the peri-ischemic region of tMCAO cortex by Nissl staining. The results revealed that SCM-198 reduced cell shrinkage and empty spaces (Figure 1(b)).

### 3.2. SCM-198 Reduced Neuron Loss after I/R Insult

**Figure 2:**
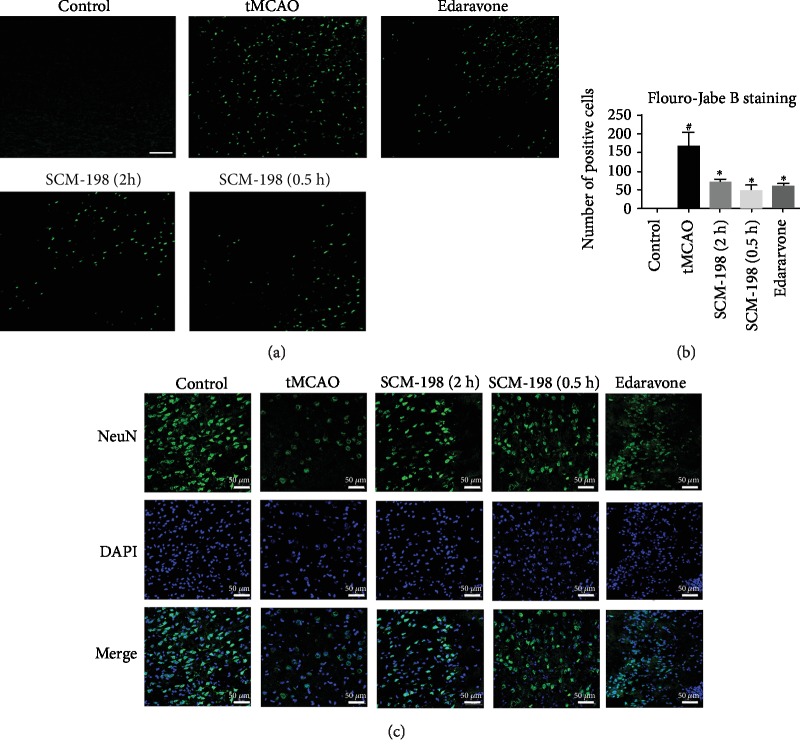
SCM-198 reduced neuron loss after I/R insult. (a) FJB staining of brain sections after reperfusion. Representative pictures of FJB staining of brain sections after reperfusion. No FJB-positive cells were found in the control group. Vast degenerating neurons were detected in the peri-ischemic regions of the tMCAO group. SCM-198 significantly reduced the number of degenerating neurons. (b) The quantitative analysis of the number of degenerating neurons. Scale bar = 50 *μ*m. Values are expressed as mean ± SD. ^#^*p* < 0.05 versus control group, ^∗^*p* < 0.05 versus tMCAO group (*n* = 5). (c) Immunofluorescence staining for NeuN after ischemia reperfusion. SCM-198 could reduce neuron loss in the ipsilateral brain cortex. Scale bar = 50 *μ*m.

Fluoro-Jade B, a kind of cell death marker used for staining degenerating neurons, was chosen for further demonstration of neuroprotection. No FJB-positive cells were found in the control group. On the contrary, vast degenerating neurons were detected in the peri-ischemic regions of the tMCAO group. SCM-198, in the 0.5 h and 2 h post operation treatment groups, significantly reduced the number of degenerating neurons. Edaravone also decreased the degenerating neurons; the effect was a little weaker than the SCM-198 0.5 h group but there was no significant difference (Figures 2(a) and 2(b)). This result was confirmed by NeuN immunoreactivity (Figure 2(c)); from the result we found that there was a substantial amount of NeuN-positive cells in the control group. tMCAO led to more neuron loss, while SCM-198 could reduce neuron loss in the ipsilateral brain cortex. There was also no significant difference between SCM-198 and edaravone. With this, these results demonstrated that SCM-198 could significantly protect against ischemic injury and improve neuronal survival.

### 3.3. SCM-198 Improved bEnd.3 Cells Antioxidative Capacity *In Vitro*

**Figure 3:**
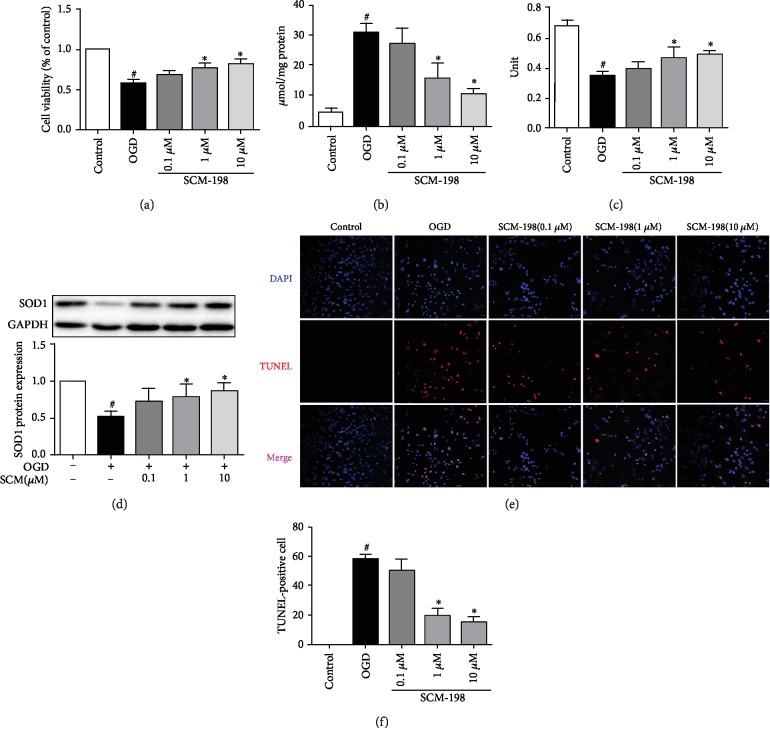
SCM-198 improved bEnd.3 cell antioxidative capacity *in vitro*. SCM-198 exhibited antioxidant abilities and improved the cell viability of bEnd.3. (a) Cell viability, evaluated by an MTT assay, was significantly reduced after OGD/R injury exposure, while 1 *μ*M and 10 *μ*M of SCM-198 could increase cell viability. (b) MDA level of the SCM-198 group was remarkably decreased as compared to the OGD/R group. (c) SCM-198 could predominantly increase intercellular antioxidative capacity by restoring the CAT activity. (d) SCM-198 obviously improved the expression of SOD1. (e) SCM-198 reduced the cell apoptosis in bEnd.3. OGD/R-induced cell apoptosis was determined by TUNEL staining. (f) The result showed that OGD/R obviously increased the apoptosis ratio, whereas treatment with SCM-198 reduced cell apoptosis. Values are expressed as mean ± SD. ^#^*p* < 0.05 versus control group, ^∗^*p* < 0.05 versus OGD group (*n* = 3).

No obvious cytotoxicity was observed at concentrations from 0.001 to 100 *μ*M SCM-198 [26]. Possible antioxidative mechanisms of SCM-198 were studied mainly using bEnd.3 cells *in vitro*. To elucidate the involvement of SCM-198 on OGD/R-induced cellular injury, the content of MDA, activity of CAT, and SOD1 expression were measured. As shown in Figure 3(a), cell viability, evaluated by an MTT assay, was significantly reduced after exposure to OGD for 6 h and reperfusion for 2 h, while SCM-198 could increase cell viability in a concentration-dependent manner. OGD/R led to cell viability decrease to 57.56% ± 3.47; 1 and 10 *μ*M of SCM-198 improve the viability to 76.54% ± 4.15 and 81.73% ± 5.18, respectively. The MDA level of the SCM-198 (1 and 10 *μ*M) group was significantly decreased as compared to the OGD/R group (Figure 3(b)). The level of MDA in the OGD/R group was two- and threefold than SCM-198 (1 and 10 *μ*M). The dose 1 and 10 *μ*M of SCM-198 could predominantly increase intercellular antioxidative capacity by restoring the activity of CAT (Figure 3(c)) and increase the expression of SOD1 (Figure 3(d)). SCM-198, 1 and 10 *μ*M, could enhance the activity of CAT from 0.35 U ± 0.03 to 0.47 U ± 0.07 and 0.49 U ± 0.03. OGD/R-induced cell apoptosis was determined by TUNEL staining; the result showed that OGD/R obviously increased the apoptosis ratio about 58.36% ± 2.72, whereas treatment with SCM-198 (1 and 10 *μ*M) inhibited cell apoptosis to 19.56% ± 4.50 and 14.70% ± 3.47 (Figure 3(e)).

### 3.4. SCM-1 98 Protected Neurons via Modulating BMECs in BMEC/Neuron Coculture System

**Figure 4:**
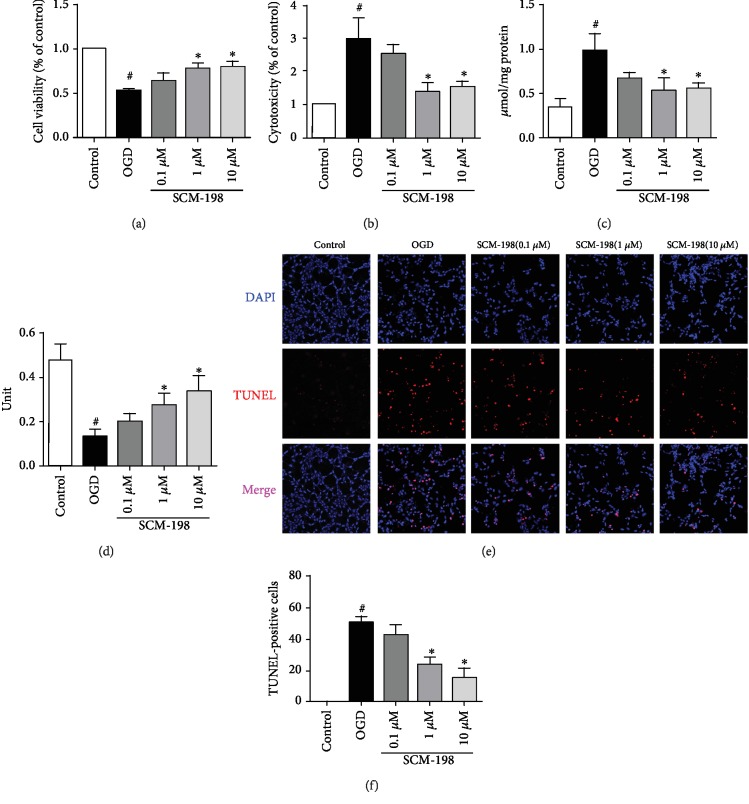
SCM-198 protected neurons via modulating BMECs in BMEC/neuron coculture system. bEnd.3 treated with SCM-198 and cocultured with SH-SY5Y exhibited protection against OGD/R injury by improving the cell viability and the antioxidative ability. (a) Treatment with SCM-198, especially 1 *μ*M and 10 *μ*M, increased bEnd.3 cell viability in OGD/R irritation. (b) SCM-198 could reduce the LDH leakage in SH-SY5Y cells. (c) SCM-198 decreased the production of MDA in SH-SY5Y cells after OGD/R injury. (d) SCM-198 increased the activity of CAT. (e) SCM-198 reduced cell apoptosis in SH-SY5Y. OGD/R-induced cell apoptosis was determined by TUNEL staining; the result showed that OGD/R obviously increased the number of apoptosis, whereas treatment with SCM-198 reduced cell apoptosis. (f) The quantitative analysis of apoptotic cells was calculated. Values are expressed as mean ± SD. ^#^*p* < 0.05 versus control group, ^∗^*p* < 0.05 versus OGD group (*n* = 3).

As SCM-198 could effectively protect against OGD/R insult in BMEC cells, we then utilized a coculture system to determine whether SCM-198 has an effect on neurons through protecting the BMECs. After 4 h reperfusion, bEnd.3 cells were cocultured with the SH-SY5Y cell line for 24 h before SH-SY5Y was subjected to OGD for 9 h and reperfusion for 2 h. bEnd.3 treatment with SCM-198 coculture with SH-SY5Y exhibited protection against OGD/R injury by improving the cell viability and antioxidant ability and reducing apoptosis.  Figure 4(a) shows that conditioned medium with SCM-198, especially 1 *μ*M and 10 *μ*M, increased the cell viability to 77.52% ± 5.84 and 80.09% ± 5.42, respectively, when compared with the OGD/R group without SCM-198 (52.95% ± 1.85). SCM-198 could reduce the LDH leakage and the MDA level in the SH-SY5Y cells and increase the activity of CAT (Figures 4(b)–4(d)). The leakage of LDH and MDA level in the OGD/R group was three times larger than the control group, while SCM-198 (1 *μ*M and 10 *μ*M) was nearly half of the OGD/R group. SCM-198, 1 *μ*M and 10 *μ*M, increased CAT activity by about 50% compared with the model group. SCM-198 could markedly decrease cell apoptosis, which was confirmed by TUNEL stain.  Figure 4(e) shows that OGD/R increased the apoptosis ratio to 50.51% ± 3.59, whereas treatment with SCM-198 (1 and 10 *μ*M) dropped down cell apoptosis to 23.12% ± 4.59 and 14.36% ± 6.53. Consistent with these observations, we believe that SCM-198 could exert a protective effect on neurons via modulating BMECs.

### 3.5. The Mechanism of SCM-198 Inhibited Apoptosis Induced by OGD/R

**Figure 5:**
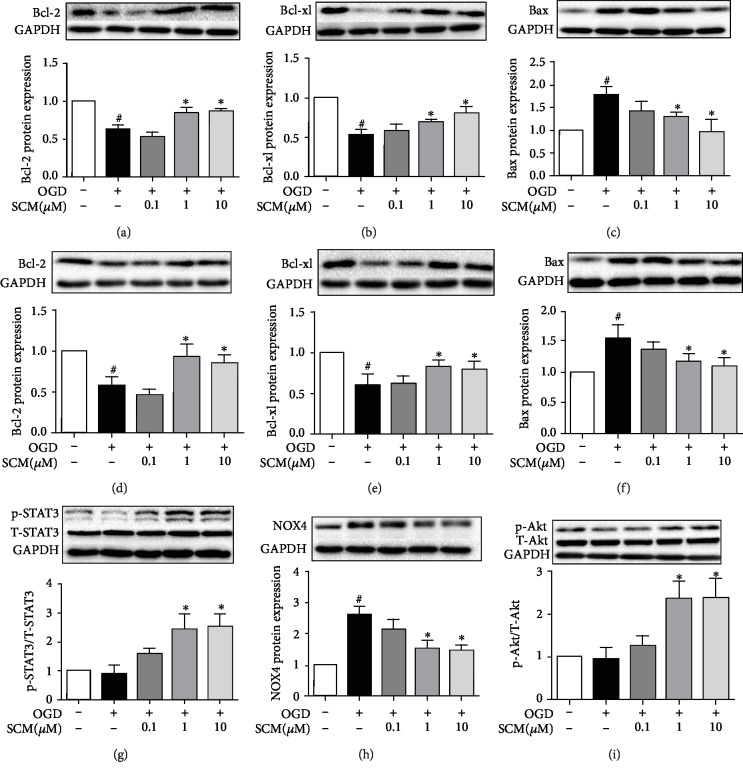
The mechanism of SCM-198 inhibited apoptosis induced by OGD/R. (a–c) SCM-198 regulated the expression of apoptosis-related protein in bEnd.3. OGD/R increased the expression of Bax. OGD/R also decreased the expression of Bcl-2 and Bcl-xl, while SCM-198 could markedly improve the expression of Bcl-2 and Bcl-xl and reduce Bax expression. (d, e) SCM-198 regulated the expression of apoptosis-related protein in SH-SY5Ycells. OGD/R increased the expression of Bax and decreased the expression of Bcl-2 and Bcl-xl, while SCM-198 could obviously improve the expression of Bcl-2 and Bcl-xl and reduce Bax expression. (g–i) SCM-198 regulated the expression of p-STAT3, NOX4, and p-Akt in bEnd.3. SCM-198 protected against apoptosis through improving the level of p-STAT3 and inhibiting the expression of NOX4, then modulated p-Akt. Values are expressed as mean ± SD. ^#^*p* < 0.05 versus control group, ^∗^*p* < 0.05 versus OGD group (*n* = 3).

Apoptosis is mainly responsible for cell death after ischemia. As we mentioned above, SCM-198 could reduce neuron loss *in vivo* and cell apoptosis *in vitro*. We examined the effect of SCM-198 on the Bcl-2 family, including the antiapoptosis protein Bcl-2 and Bcl-xl and proapoptosis protein Bax. Our results showed that following OGD/R injury Bcl-2 and Bcl-xl significantly decreased, whereas they were improved with SCM-198 treatment (1 and 10 *μ*M) (Figures 5(a)–5(c)). At the same time, the coculture results are consistent with the findings in bEnd.3 cells. BMEC treatment with SCM-198 cocultured with SH-SY5Y exerted protection against apoptosis induced by OGD/R by increasing the expression of Bcl-2 and Bcl-xl and reducing the Bax level (Figures 5(d)–5(f)).

Next, we further explored the mechanism of SCM-198 in reducing cell apoptosis caused by OGD/R in bEnd.3 cells. The results indicated that SCM-198, 1 and 10 *μ*M, protected against apoptosis through improving the level of p-STAT3 and inhibiting the expression of NOX4, then modulated p-Akt, the proteins which were involved in cell apoptosis (Figures 5(g)–5(i)).

### 3.6. SCM-198 Inhibited Apoptosis through the STAT3/NOX4/Bcl-2 Pathway

**Figure 6:**
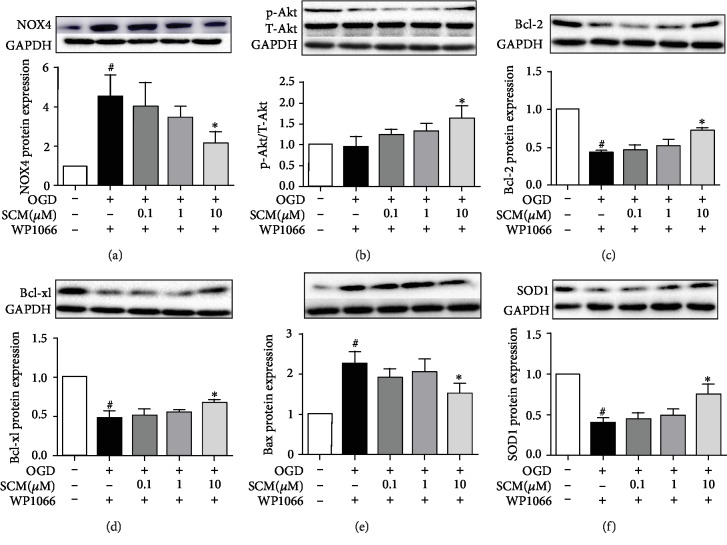
SCM-198 inhibited apoptosis through the STAT3/NOX4/Bcl-2 pathway. (a) SCM-198 (10 *μ*M) still observably decreased the overexpression of NOX4 induced by WP1066. (b) SCM-198 (10 *μ*M) improved the expression of p-Akt. (c–e) SCM-198 (10 *μ*M) could enhance the protection against apoptosis after inhibiting the activation of p-STAT3 in bEnd.3. (f) SCM-198 (10 *μ*M) improved the expression of SOD1 after using WP1066. Values are expressed as mean ± SD. ^#^*p* < 0.05 versus control group, ^∗^*p* < 0.05 versus OGD group (*n* = 3).

As we know, STAT3 and NOX4 are both involved in regulating apoptosis by modulating the PI3K/Akt pathway, but the connection between STAT3 and NOX4 remains unclear. Firstly, we used IL-6 to upregulate p-STAT3 in different concentrations or WP1066 to inhibit STAT3 1 h before OGD/R injury; western blot indicated that the level of NOX4 was inhibited by the overexpression of p-STAT3 and increased by inhibiting STAT3, respectively ([Supplementary-material supplementary-material-1]). But when we used DPI or GKT137831 to inhibit NOX4 before being subjected to OGD/R, the level of STAT3 was unchanged ([Supplementary-material supplementary-material-1]). We deemed that STAT3 could regulate the expression in ischemic stroke, so we used WP1066 for further study. The results revealed that treatment with 10 *μ*M of SCM-198 still observably decreased the overexpression of NOX4 induced by WP1066 and improved the expression of p-Akt. Then, SCM-198 further reduced the level of Bax and increased the expression of Bcl-xl and Bcl-2 (Figure 6).

### 3.7. SCM-198 Upregulates Interaction between p-STAT3 and NOX4

**Figure 7:**
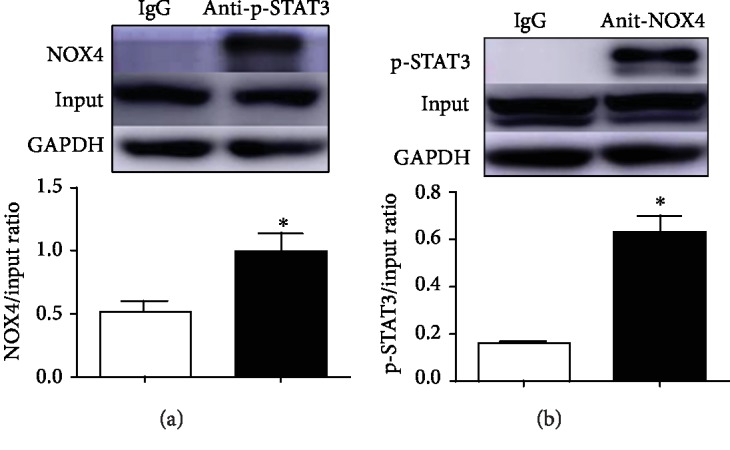
SCM-198 upregulates the interaction between p-STAT3 and NOX4. (a, b) The interactions between p-STAT3 and NOX4 were confirmed by immunoprecipitation. Values are expressed as mean ± SD. ^∗^*p* < 0.05 versus IgG group (*n* = 3).

Our previous results have indicated that p-STAT3 participated in OGD/R-mediated NOX4 expression. We speculated that SCM-198 could affect the interaction between p-STAT3 and NOX4. Coimmunoprecipitation analysis demonstrated that the interaction between p-STAT3 and NOX4 was increased by treatment with SCM-198 (Figures 7(a) and 7(b)). These data suggested that SCM-198 improved p-STAT3-NOX4 interaction, which may inhibit NOX4 activation and subsequent apoptosis.

## 4. Discussion

In the present study, we demonstrated that NOX4 and apoptosis pathway mediated the protective effects of SCM-198 on endothelial cells and neurons during stroke *in vivo* and *in vitro*. In addition, we newly discovered and elucidated the p-STAT3/NOX4 pathway influenced by SCM-198 during BBB breakdown. The expression of p-STAT3 serves as a negative regulator of NOX4, and maybe it is achieved through direct binding in this hypoxia occasion affected by SCM-198. These results provide new insights into the stroke protective roles of SCM-198 apart from BBB maintenance we have reported recently [25].

In ischemic stroke, the brain firstly suffers from a vast loss of oxygen and nutrient causing tissue damage mainly in the cortex and striatum [38], and reperfusion aggravates the insult due to the fresh oxygen [3]. Conventional remedies for stroke include tPA, an enzyme that recommended for clinical use to catalyze blood clots less than 3 hours after acute ischemia occurs, and edaravone, a free radical scavenger and the only neuroprotective agent clinically approved for acute ischemic stroke in Japan [39]. However, studies have reported that treatment with tPA is frequently accompanied with a detrimental complication such as brain edema because of reperfusion pursued. Edaravone could suppress ROS production and potentially suppress the open of mitochondrial permeability transition pore (MPTP) [39]. Until now, edaravone is the only clinically approved treatment for stroke in Japan and treatment for amyotrophic lateral sclerosis (ALS) in the US and Japan. The limited availability of effective clinical medicine leads to a large unmet need in society, so the development of new approaches for acute stroke management is urgent.

SCM-198 has been reported to have cardioprotective effects against ischemic myocardial injuries through scavenging intracellular ROS and increasing antiapoptosis-associated protein levels [40]. In addition, several studies have reported that SCM-198 can ameliorate the infarction area of the cerebral cortex and improve neurological damage [24, 41]. In this study, we found that the administration of SCM-198 0.5 h post I/R in rat could preserve neuron morphology while neurons in the edaravone treatment group were still swelling and with less dense cytoplasm mass. In the meantime, SCM-198 could prevent neural cell loss in the peri-ischemic region of the cortex (Figure 1). Furthermore, this was repeatedly confirmed by FJB staining and NeuN detecting. The effect of SCM-198 was a little better than edaravone although there was no significance. But as we know, SCM-198 has fewer side effects and is easier to obtain. In the *in vitro* study, we induced bEnd.3 cells or coculture system with OGD/R model. The results reveal that SCM-198 significantly improved cell viability and inhibited cell apoptosis without obvious cytotoxicity in the OGD/R-induced cells. But the results displayed that the effect of SCM-198 with 1 and 10 *μ*M sometimes showed a dose response effect between bEnd.3 cells and SH-SY5Y cells. In SH-SY5Y cells, 1 and 10 *μ*M sometimes worked the same way; we speculated that there may be two reasons: (1) conditioned media influenced the results and (2) different experimental systems. Taken together, SCM-198 might play a neuroprotective role in I/R and OGR/R conditions.

STAT3 was reported to be a controversial contributor to cerebral ischemic reperfusion injury. The JAK2/STAT3 pathway is made up of JAK and STAT protein family. Among the STAT proteins, STAT3 is considered the most conserved, and it can be stimulated by various factors and stimuli [18], such as inflammatory cytokines and chemokines. There are contrary functional options about JAK2/STAT3 activation in cerebral ischemia [42]. Many previous studies are agree with that the activation of the JAK2/STAT3 pathway attenuates brain ischemia/reperfusion injury [43]. It is reported that estradiol or catalpol could protect against I/R injury through activating STAT3 [44], which is consistent with the results of ours. In order to make sure the relationship between STAT3 activation and the neuroprotective effects of SCM-198, WP1066, a STAT3 inhibitor, was utilized. Our results revealed that WP1066 could partially counteract the protective effect of SCM-198 (Figure 6), while the overexpression of p-STAT3 could inhibit the expression of NOX4. Co-IP experiment confirmed the direct binding of p-STAT3 and NOX4, and the binding activity could be regulated by SCM198. In addition, the inhibition of NOX4, expression of p-STAT3, was not influenced, indicating that NOX4 was regulated by p-STAT3.

NOX4, a primary source of ROS, is highly expressed in many tissues during hypoxia which suggested that NOX4 could be an important uniform therapeutic target for postischemic injuries. Furthermore, Kleinschnitz et al. reported that NOX4 predominantly localizes in endothelial cells and neurons in the brain (rodent and human) [12]. In the meantime, the breakdown of BBB, the special structure that differentiates the brain from the heart and other organs, could be attributed to the ROS produced by the endothelial NOX4 during ischemic stroke [13]. Neuronal NOX4 also contributes majorly to neuronal cellular autotoxicity upon ischemia or hypoxia [13]. Pharmacological inhibition of NOX4 could be a promising approach to develop stroke protective drugs. Large-animal stroke models and preparation for clinical trial are ongoing (European Research Council-Proof of Concept Project 737586 SAVEBRAIN). In our study, SCM-198 could markedly reduce the upregulation of NOX4 in endothelial cells and neuronal cells suffering from ischemic condition, which was consistent with our previous studies [21, 25].

## 5. Conclusion

**Figure 8:**
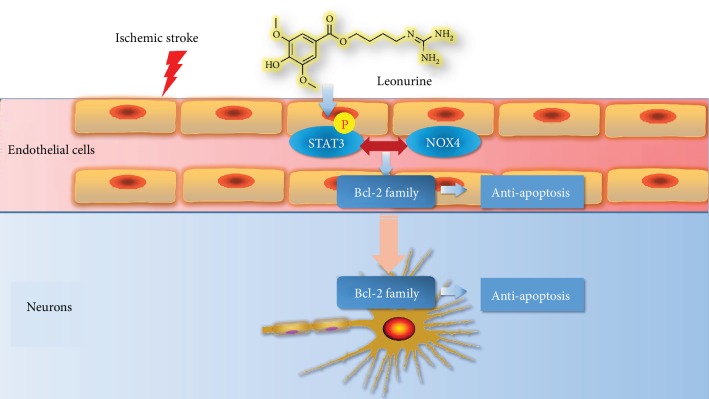
SCM-198 could be the potential drug for neuroprotective effect through stabilizing endothelial cell function.

In summary, our results showed that SCM-198 could exert neuroprotective effects by stabilizing endothelial cell function through regulating the p-STAT3/NOX4/Bcl-2 pathway (Figure 8). Moreover, the regulation of NOX4 could be due to the direct binding to p-STAT3 protein, which could be affected by SCM-198. SCM-198 could be the potential drug for I/R injury.

## Data Availability

All data generated or analyzed during this work are included in this published paper.

## Abbreviations

tPA: Tissue-type plasminogen activator
STAT3: Activated signal transducers and transcription 3
tMCAO: Transient middle cerebral artery occlusion
FJB: Fluoro-Jade B
TEM: Transmission electron microscopy
CBF: Cerebral blood flow
DAPI: 4′, 6-Diamidino-2-phenylindole
ATCC: American Type Culture Collection
FBS: Fetal bovine serum
DMEM: Dulbecco's modified Eagle's medium
GSH: Glutathione
MTT: 3-[4,5-Dimethylthiazol-2-yl]-2,5-diphenyl tetrazolium bromide
OGD/R: Oxygen-glucose deprivation and reoxygenation
MDA: Malondialdehyde
LDH: Lactate dehydrogenase
CAT: Catalase
TUNEL: Terminal deoxynucleotidyl transferase-mediated dUTP-biotin nick-end labeling
ALS: Amyotrophic lateral sclerosis
ECs: Endothelial cells
NOX: NADPH oxidases
BBB: Blood-brain barrier
MPTP: Mitochondrial permeability transition pore.

## Supplementary Materials

Supplementary Materials Supplementary1: inhibition of the activity of NOX4 had no effect on the expression of pSTAT3. The inhibitors of NOX4, GKT137831 and DPI, were used before OGD/R injury. But they could not influence the expression of p-STAT3. Supplementary2: upregulation of the expression of p-STAT3 by IL6 could inhibit the level of NOX4 in bEnd.3. A: different concentrations of IL6 were used to improve the expression of p-STAT3 after 12 h incubation; B: IL6 stimulated bEnd.3 for 12 h could not inhibit the expression of NOX4 after OGD/R injury; C: 17.5 ng/mL of IL6 significantly decreased the expression of NOX4 after incubation for 24, 48, and 72 h; D:WP1066 could exacerbate the activation of NOX4 induced by OGD/R injury.

## References

[1] Tsai C. F., Thomas B., Sudlow C. L. M. (2013). Epidemiology of stroke and its subtypes in Chinese vs white populations: a systematic review. *Neurology*.

[2] Liu J., Wang Y., Akamatsu Y. (2014). Vascular remodeling after ischemic stroke: mechanisms and therapeutic potentials. *Progress in Neurobiology*.

[3] Radermacher K. A., Wingler K., Langhauser F. (2013). Neuroprotection after stroke by targeting NOX4 as a source of oxidative stress. *Antioxidants & Redox Signaling*.

[4] Ying W., Xiong Z. G. (2010). Oxidative stress and NAD+ in ischemic brain injury: current advances and future perspectives. *Current Medicinal Chemistry*.

[5] Casas A. I., Dao V. T. V., Daiber A. (2015). Reactive oxygen-related diseases: therapeutic targets and emerging clinical indications. *Antioxidants & Redox Signaling*.

[6] Ma M. W., Wang J., Zhang Q. (2017). NADPH oxidase in brain injury and neurodegenerative disorders. *Molecular Neurodegeneration*.

[7] Lassegue B., San Martin A., Griendling K. K. (2012). Biochemistry, physiology, and pathophysiology of NADPH oxidases in the cardiovascular system. *Circulation Research*.

[8] Maejima Y., Kuroda J., Matsushima S., Ago T., Sadoshima J. (2011). Regulation of myocardial growth and death by NADPH oxidase. *Journal of Molecular and Cellular Cardiology*.

[9] Doerries C., Grote K., Hilfiker-Kleiner D. (2007). Critical role of the NAD(P)H oxidase subunit p47phox for left ventricular remodeling/dysfunction and survival after myocardial infarction. *Circulation Research*.

[10] Craige S. M., Chen K., Pei Y. (2011). NADPH oxidase 4 promotes endothelial angiogenesis through endothelial nitric oxide synthase activation. *Circulation*.

[11] Kleikers P. W., Hooijmans C., Göb E. (2015). A combined pre-clinical meta-analysis and randomized confirmatory trial approach to improve data validity for therapeutic target validation. *Scientific Reports*.

[12] Kleinschnitz C., Grund H., Wingler K. (2010). Post-stroke inhibition of induced NADPH oxidase type 4 prevents oxidative stress and neurodegeneration. *PLoS Biology*.

[13] Casas A. I., Geuss E., Kleikers P. W. M. (2017). NOX4-dependent neuronal autotoxicity and BBB breakdown explain the superior sensitivity of the brain to ischemic damage. *Proceedings of the National Academy of Sciences of the United States of America*.

[14] Liu X., Zhang X., Zhang J. (2014). Diosmin protects against cerebral ischemia/reperfusion injury through activating JAK2/STAT3 signal pathway in mice. *Neuroscience*.

[15] Chen G., Zhang S., Shi J., Ai J., Hang C. (2009). Effects of recombinant human erythropoietin (rhEPO) on JAK2/STAT3 pathway and endothelial apoptosis in the rabbit basilar artery after subarachnoid hemorrhage. *Cytokine*.

[16] Zhu H., Zou L., Tian J., du G., Gao Y. (2013). SMND-309, a novel derivative of salvianolic acid B, protects rat brains ischemia and reperfusion injury by targeting the JAK2/STAT3 pathway. *European Journal of Pharmacology*.

[17] Raible D. J., Frey L. C., Brooks-Kayal A. R. (2014). Effects of JAK2-STAT3 signaling after cerebral insults. *JAK-STAT*.

[18] Liang Z., Wu G., Fan C. (2016). The emerging role of signal transducer and activator of transcription 3 in cerebral ischemic and hemorrhagic stroke. *Progress in Neurobiology*.

[19] Amantea D., Tassorelli C., Russo R. (2011). Neuroprotection by leptin in a rat model of permanent cerebral ischemia: effects on STAT3 phosphorylation in discrete cells of the brain. *Cell Death & Disease*.

[20] Satriotomo I., Bowen K. K., Vemuganti R. (2006). JAK2 and STAT3 activation contributes to neuronal damage following transient focal cerebral ischemia. *Journal of Neurochemistry*.

[21] Liu X. H., Pan L. L., Deng H. Y. (2013). Leonurine (SCM-198) attenuates myocardial fibrotic response via inhibition of NADPH oxidase 4. *Free Radical Biology & Medicine*.

[22] Chen C. X., Kwan C. Y. (2001). Endothelium-independent vasorelaxation by leonurine, a plant alkaloid purified from Chinese motherwort. *Life Sciences*.

[23] Liu X. H., Chen P. F., Pan L. L., Silva R. D., Zhu Y. Z. (2009). 4-Guanidino-n-butyl syringate (Leonurine, SCM 198) protects H9c2 rat ventricular cells from hypoxia-induced apoptosis. *Journal of Cardiovascular Pharmacology*.

[24] Loh K. P., Qi J., Tan B. K. H., Liu X. H., Wei B. G., Zhu Y. Z. (2010). Leonurine protects middle cerebral artery occluded rats through antioxidant effect and regulation of mitochondrial function. *Stroke*.

[25] Zhang Q. Y., Wang Z. J., Sun D. M. (2017). Novel therapeutic effects of leonurine on ischemic stroke: new mechanisms of BBB integrity. *Oxidative Medicine and Cellular Longevity*.

[26] Hong Z. Y., Shi X. R., Zhu K., Wu T. T., Zhu Y. Z. (2014). SCM-198 inhibits microglial overactivation and attenuates A*β*1-40-induced cognitive impairments in rats via JNK and NF-кB pathways. *Journal of Neuroinflammation*.

[27] Ortega F. J., Jolkkonen J., Mahy N., Rodríguez M. J. (2013). Glibenclamide enhances neurogenesis and improves long-term functional recovery after transient focal cerebral ischemia. *Journal of Cerebral Blood Flow and Metabolism*.

[28] Sun K., Hu Q., Zhou C. M. (2010). Cerebralcare Granule^®^, a Chinese herb compound preparation, improves cerebral microcirculatory disorder and hippocampal CA1 neuron injury in gerbils after ischemia-reperfusion. *Journal of Ethnopharmacology*.

[29] Wei C. C., Kong Y. Y., Hua X. (2017). NAD replenishment with nicotinamide mononucleotide protects blood–brain barrier integrity and attenuates delayed tissue plasminogen activator‐induced haemorrhagic transformation after cerebral ischaemia. *British Journal of Pharmacology*.

[30] Liu F., Schafer D. P., McCullough L. D. (2009). TTC, fluoro-Jade B and NeuN staining confirm evolving phases of infarction induced by middle cerebral artery occlusion. *Journal of Neuroscience Methods*.

[31] Pan L. L., Liu X. H., Shen Y. Q. (2013). Inhibition of NADPH oxidase 4-related signaling by sodium hydrosulfide attenuates myocardial fibrotic response. *International Journal of Cardiology*.

[32] Xin W., Huang C., Zhang X. (2014). Methyl salicylate lactoside inhibits inflammatory response of fibroblast‐like synoviocytes and joint destruction in collagen‐induced arthritis in mice. *British Journal of Pharmacology*.

[33] Liu J., Jin X., Liu K. J., Liu W. (2012). Matrix metalloproteinase-2-mediated occludin degradation and caveolin-1-mediated claudin-5 redistribution contribute to blood-brain barrier damage in early ischemic stroke stage. *The Journal of Neuroscience*.

[34] Wang J., Chen Y., Yang Y. (2016). Endothelial progenitor cells and neural progenitor cells synergistically protect cerebral endothelial cells from hypoxia/reoxygenation-induced injury via activating the PI3K/Akt pathway. *Molecular Brain*.

[35] Lv G., Sun D., Zhang J. (2017). Lx2-32c, a novel semi-synthetic taxane, exerts antitumor activity against prostate cancer cells _in vitro_ and _in vivo_. *Acta Pharmaceutica Sinica B*.

[36] Liu X., Pan L., Wang X., Gong Q., Zhu Y. Z. (2012). Leonurine protects against tumor necrosis factor-*α*-mediated inflammation in human umbilical vein endothelial cells. *Atherosclerosis*.

[37] Du G., Zhu H., Yu P. (2013). SMND-309 promotes angiogenesis in human umbilical vein endothelial cells through activating erythropoietin receptor/STAT3/VEGF pathways. *European Journal of Pharmacology*.

[38] Yang Y., Estrada E. Y., Thompson J. F., Liu W., Rosenberg G. A. (2007). Matrix metalloproteinase-mediated disruption of tight junction proteins in cerebral vessels is reversed by synthetic matrix metalloproteinase inhibitor in focal ischemia in rat. *Journal of Cerebral Blood Flow and Metabolism*.

[39] Matsumoto S., Murozono M., Kanazawa M., Nara T., Ozawa T., Watanabe Y. (2018). Edaravone and cyclosporine A as neuroprotective agents for acute ischemic stroke. *Acute Medicine & Surgery*.

[40] Liu X. H., Pan L. L., Chen P. F., Zhu Y. Z. (2010). Leonurine improves ischemia-induced myocardial injury through antioxidative activity. *Phytomedicine*.

[41] Qi J., Hong Z. Y., Xin H., Zhu Y. Z. (2010). Neuroprotective effects of leonurine on ischemia/reperfusion-induced mitochondrial dysfunctions in rat cerebral cortex. *Biological & Pharmaceutical Bulletin*.

[42] Hu G., Yang T., Zhou J. (2017). Mechanism of surrounding rock failure and crack evolution rules in branched pillar recovery. *Minerals*.

[43] Chen H., Lin W., Zhang Y. (2016). IL-10 promotes neurite outgrowth and synapse formation in cultured cortical neurons after the oxygen-glucose deprivation via JAK1/STAT3 pathway. *Scientific Reports*.

[44] Sehara Y., Sawicka K., Hwang J. Y., Latuszek-Barrantes A., Etgen A. M., Zukin R. S. (2013). Survivin is a transcriptional target of STAT3 critical to estradiol neuroprotection in global ischemia. *The Journal of Neuroscience*.

