# Supplementary material for: Neuroprotective Effect of SCM-198 through Stabilizing Endothelial Cell Function
Source: Oxid Med Cell Longev. 2019 Nov 11;2019:7850154. doi: 10.1155/2019/7850154 (PMC6885260; doi:10.1155/2019/7850154)

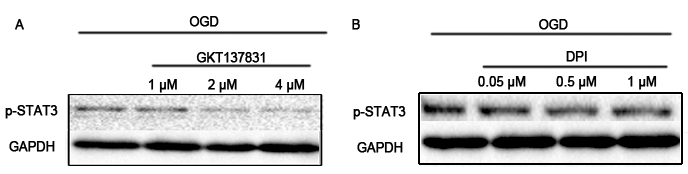


**Supplementary1. Inhibiting the activity of NOX4 had no effect on the expression of pSTAT3.** The inhibitors of NOX4, GKT137831, and DPI, were used before OGD/R injury. But they could not influence the expression of p-STAT3.


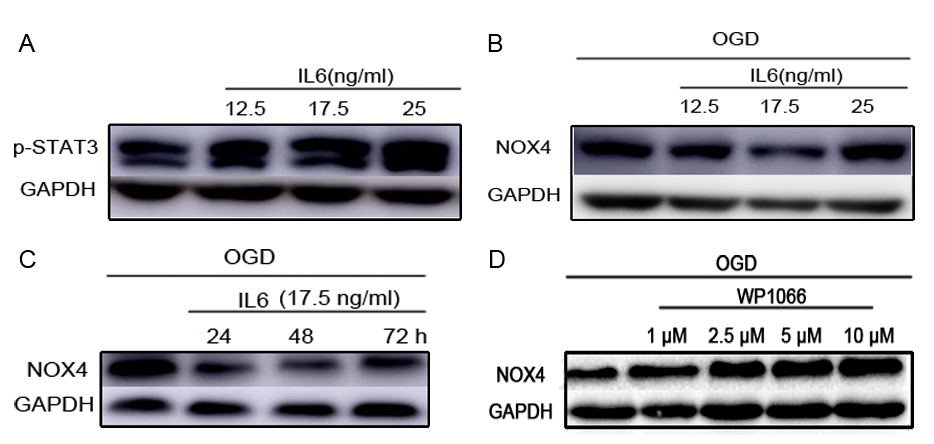


**Supplementary2. Upregulated the expression of p-STAT3 by IL6 could inhibit the level of NOX4 in bEnd.3.** A: Different concentration of IL6 were used to improve the expression of p-STAT3 after 12 h incubation; B: IL6 stimulated bEnd.3 for 12 h could not inhibit the expression of NOX4 after OGD/R injury; C: 17.5 ng/mL of IL6 significantly decreased the expression of NOX4 after incubation for 24, 48 and 72 h; D:WP1066 could exacerbate the activation of NOX4 induced by OGD/R injury.

**un-cropped images of the original western blots**

**b.End3 cell**

SOD1


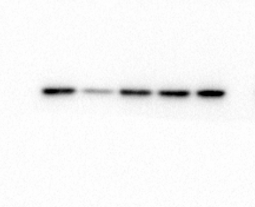


Bcl-2


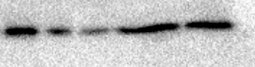


Bcl-2+WP1066


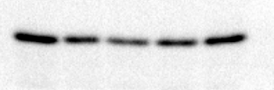


Bcl-xl


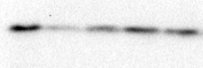


Bcl-xl+WP1066


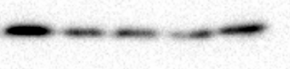


Bax


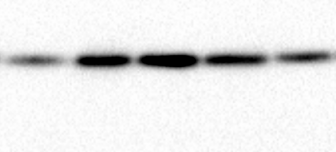


Bax+WP1066


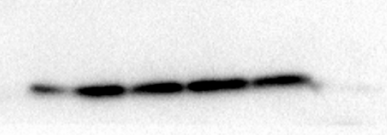


p-STAT3


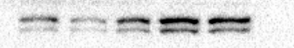


T-Stat3


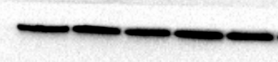


NOX4


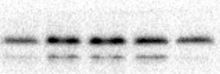


NOX4+WP1066


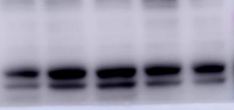


p-Akt


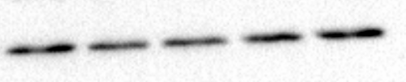


T-Akt


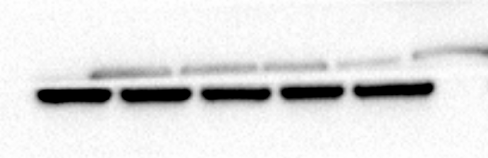


P-Akt+WP1066


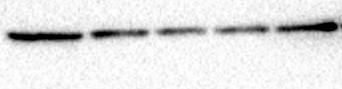


T-Akt+WP1066


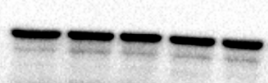


**SH-SY5Y Cell**

Bax


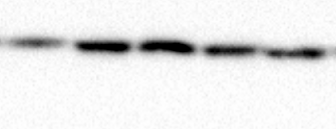


Bcl-2


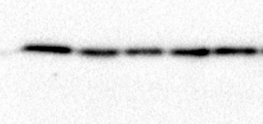


Bcl-xl


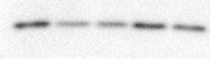

Supplement: Supplementary Materials — Supplementary1: inhibition of the activity of NOX4 had no effect on the expression of pSTAT3. The inhibitors of NOX4, GKT137831 and DPI, were used before OGD/R injury. But they could not influence the expression of p-STAT3. Supplementary2: upregulation of the expression of p-STAT3 by IL6 could inhibit the level of NOX4 in bEnd.3. A: different concentrations of IL6 were used to improve the expression of p-STAT3 after 12 h incubation; B: IL6 stimulated bEnd.3 for 12 h could not inhibit the expression of NOX4 after OGD/R injury; C: 17.5 ng/mL of IL6 significantly decreased the expression of NOX4 after incubation for 24, 48, and 72 h; D:WP1066 could exacerbate the activation of NOX4 induced by OGD/R injury. [file 7850154.f2.docx]
